# A Versatile Illumination System for Real-Time Terahertz Imaging

**DOI:** 10.3390/s20143993

**Published:** 2020-07-17

**Authors:** Jean-Baptiste Perraud, Adrien Chopard, Jean-Paul Guillet, Pierre Gellie, Antoine Vuillot, Patrick Mounaix

**Affiliations:** 1 IMS—Bordeaux University, UMR CNRS 5218, Bât A31, 351 Cours de la Libération, 33400 Talence, France; jean-baptiste.perraud@u-bordeaux.fr (J.-B.P.); adrien.chopard@u-bordeaux.fr (A.C.); jean-paul.guillet@u-bordeaux.fr (J.-P.G.); 2 Lytid—8 rue la Fontaine, 92120 Montrouge, France; p.gellie@lytid.com (P.G.); a.vuillot@lytid.com (A.V.)

**Keywords:** terahertz, imaging, versatility, illumination, microbolometer, lissajous, real-time, non-destructive-testing, tomography

## Abstract

Terahertz technologies are attracting strong interest from high-end industrial fields, and particularly for non-destructive-testing purposes. Currently lacking compactness, integrability as well as adaptability for those implementations, the development and commercialisation of more efficient sources and detectors progressively ensure the transition toward applicative implementations, especially for real-time full-field imaging. In this work, a flexible illumination system, based on fast beam steering has been developed and characterized. Its primary goal is to suppress interferences induced by the coherence length of certain terahertz sources, spoiling terahertz images. The second goal is to ensure an enhanced signal-to-noise ratio on the detector side by the full use and optimized distribution of the available power. This system provides a homogeneous and adjustable illumination through a simplified setup to guarantee optimum real-time imaging capabilities, tailored to the sample under inspection. Working toward industrial implementations, different illumination process are conveniently assessed as a result of the versatility of this method.

## 1. Introduction

For more than a decade, terahertz imaging has demonstrated its ability to detect, localize or identify compounds inside optically opaque materials since some dielectric materials, like polymers or ceramics, have relatively low absorption coefficients in this part of the electromagnetic spectrum. Compared to other techniques, like ultrasounds or X-ray imaging, terahertz imaging represents a contact-free and harmless solution, suitable for many different potential non-destructive testing (NDT) applications such as maintenance or manufacturing industry of composite material [1,2], food quality inspection [3,4], art conservation and authentification [5,6] or for biomedical sciences [7,8]. Terahertz imaging can also be used as an efficient and alternative tool for metrology, allowing length extraction or surface and volume inspection [9]. Moreover, pushing toward more complex implementations, considering different projections of the same object, 3D terahertz reconstructions have been demonstrated, either by computed tomography [10], or through a defocus measurement [11]. However, unlike optical microscopy or infrared imaging that take advantages of a more advanced technological maturity with the uses of matrix sensors, most of terahertz images are performed through a slow and laborious raster-scanning process in a controlled environment, ensuring high Signal to Noise Ratio (SNR) through a basic implementation. Nevertheless, this technique remains extremely time-consuming to obtain a relevant graphical depiction [12], requiring several hours of measurements for a 2D image and up to days to complete the stack of images required for a 3D tomographic reconstruction [10]. This tedious process is far from matching with the required frame-rate targeted for quality control or non-destructive testing applications in an industrial context. To face this limitation, research groups and cutting-edge technology centers have developed and are commercialising a variety of compact and uncooled terahertz matrix sensors based on several technological advances, from micro-bolometer integration to semi-conductors hetero-structures [13,14,15,16], in order to cover different frequency bands from 100 GHz up to 5 THz. The use of residual sensitivity of Long Wave InfraRed (LWIR) cameras in the terahertz domain has also been assessed [17].

Without any mechanical displacement, those technologies can provide images at video rates [18,19,20,21]. However, despite high-sensitivity sensors and terahertz radiation optimized lenses, the full-field technique does not achieve object-representative imaging as properly as raster-scanning for three main reasons. First, scaling from a single diffraction-limited spot for raster scanning up to the full surface of several sensors to be illuminated, the power density decrease leads to a drastically lowered image average SNR. Along with the limited power of compact sources in the terahertz domain, up to few milliwatts, this SNR loss may lead to extremely noisy or irrelevant images. Secondly, induced by the Gaussian-like beam profile, the object field is exposed to a non-uniform radiation power, and the potential SNR in the vicinity of the optical axis generally reaches higher values than for off-axis contributions. Finally, due to the sources high coherency and the optical path differences through the sample and the lens, strong interference artifacts appear in the sensor plane and irrevocably degrade the image quality [22]. In order to overcome the last limiting factor, one solution consists in quickly modifying the optical path by using wobbling mirrors. Considering the relatively long time response of the sensors, a fast enough variation of the interference fringes on the image plane will lead to an averaging and smoothing of those fringes on the recorded output signal. Such a random optical path variation by a reflecting or scattering optical scrambler can limit the effect of the aforementioned interference pattern on the final image. This technique has been investigated for laser projection application in order to average out what is called speckle in the visible range, an interference pattern induced by the diffusion of wavelength-sized particles [23]. This solution has also been investigated in the terahertz range, demonstrating an improved illumination quality, but inducing a significant radiation power loss [24].

Another more robust solution to overcome this coherency issues consists in fast assessing the illumination beam position, using galvanometric beam steering to ensure averaging over imaging frames. This solution has already been investigated for sensors array characterisation [25], and has shown an improved beam homogenisation. We propose to further explore this solution at 2.5 THz and 3.78 THz, not only to limit the impact of the interference fringes on the image, but also to implement an enhanced control over the illuminated area, better suiting the size and the opacity of inspected objects, for an improved versatility and adaptability in real-time imaging applications.

## 2. Experimental Implementation

**Figure 1:**
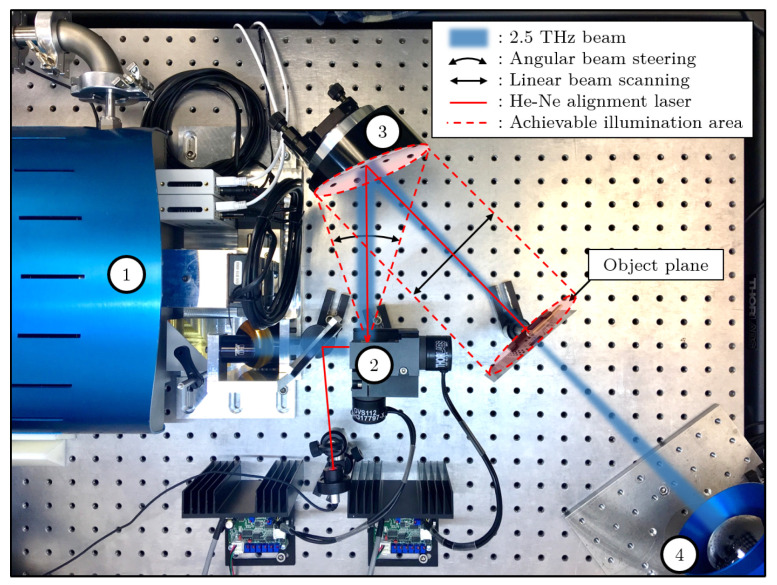
Experimental imaging setup, implementing (1) the Teracascade1000 QCL source, from Lytid, emitting 1.3 mW at 2.5 THz, (2) the double mirror galvanometer, (3) a 45${}^{{}^{\circ}}$ off-axis parabolic mirror and (4) the INO 288 × 384 microbolometer array with an aspherical f/0.8 addapted silicon lens, Teralens from Lytid.

Targeting real-time imaging improvement for NDT applications, the experimental setup is based on a full-field imaging system. It includes a compact terahertz Quantum Cascade Laser (QCL) as light source, and an uncooled microbolometer sensors array as a detector. For the first imaging system (Figure 1), the microbolometer array from the INO IRXCAM (240 × 320 pixels with 35 μm pitch), combined with an aspherical f/0.8 and 0.22 magnification silicon lens with 20 cm working distance, is illuminated by the QCL TC1000 from Lytid SAS, emitting 1.3 mW at 2.5 THz combined to its dedicated auto-alignment module. A second imaging setup have also been investigated, implementing the TZcam LETI microbolometer array (240 × 320 pixels with 50 μm pitch) combined with its f/0.8 and 0.25 magnification silicon lens, and illuminated by the TC100 QCL, emitting 0.1 mW at 3.78 THz. In order to overcome the limiting artifacts induced by the high coherence of such THz QCL sources, and develop the versatility of the imaging system, the homogeneous illumination on the object plane is performed via a combination of fast beam steering and adequate imaging exposure, thanks to the implementation of a commercially available double mirror galvanometer. Allowing a controllable angular beam steering up to ± 20° along both x and y directions with a maximum operating frequency of 130 Hz, each mirror is mounted on a voltage commanded motor, fed by a dedicated controller. A variety of voltage waveforms with adjustable offsets and amplitudes are thus achievable, in favor of the versatility of the system. The second mirror of the galvanometer is located at the focal point of a 45° off-axis parabolic mirror OAPM with 4″ diameter and 152.4 mm parent focal length, converting the inefficient angular beam steering in an appropriate linear beam scanning toward the object plane and camera. Both lighting experimental setups start from a THz QCL diverging beam, shaped into a 10 mm diameter collimated beam for a proper injection into the galvanometer. For alignment and centering purposes as well as illumination pattern visualisation, the two experimental imaging systems feature a He–Ne alignment laser diode following the THz beam thanks to a 2 μm thick THz-transparent membrane.

**Figure 2:**
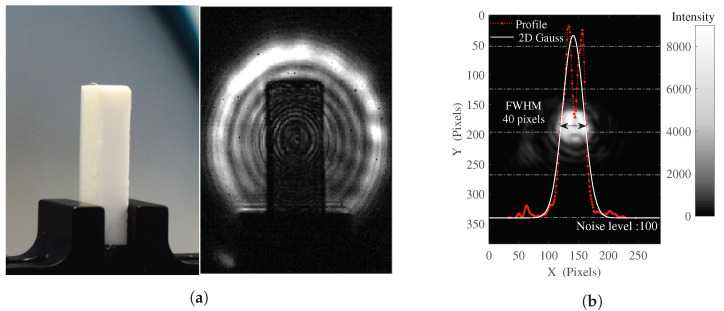
(**a**) Typical transmission image at 2.5 THz of a phase object (PTFE) illuminated by a simple diverging beam from a coherent emitter. (**b**) Image, profile and 2D Gaussian fit profile of the terahertz beam resulting from the newly introduced experimental setup in still configuration.

Demonstrating the problematic aspect of the light source’s coherence when it comes to imaging capabilities, Figure 2a displays a typical image in transmission mode at 2.5 THz, obtained from an illumination system where no specific precautions have been taken. The interference patterns from the illumination source are clearly observable, and eradicate the imaging and tomographic reconstruction capabilities of such a simple system, by inducing strong coherence related artifacts when interacting with the illuminated object, a parallelepiped of Teflon in this case. No corrective processing are practicable since the recorded inner interferogram is highly impacted by the geometry of the object itself, preventing the use of a reference background recording. Besides, because of the shift of the interference fringes when displacing or rotating the object, as shown in the Supplementary Video S1, no correction algorithms would lead to any relevant object reconstruction.

In this newly introduced illumination system, the beam itself is strongly impacted by this light source coherence characteristic as well, as depicted in Figure 2b, where an image, a beam profile cut and the related 2D Gaussian fit profile are plotted, resulting in an estimated FWHM *H* = 40 pixels, 1.40 mm in the imaging plane, or 6.4 mm in the object plane.

Nevertheless, in order to overcome those profile irregularities and reach a homogeneous illumination, the mirror wobbling should average the induced coherency artifacts when steering the beam over the object plane fast enough in comparison to the camera frame rate. To properly fulfill its task, in wobbling mode, the galvanometer should be set with high enough angular oscillation frequencies ($F_{x}$ and $F_{y}$), as to ensure an averaged recording of the varying radiation power over the microbolometer array integration time. Intrinsically, the maximum camera frame rate, 50 Frames Per Second (FPS) for INO IRXCAM (20 ms integration time) and 25 FPS for the TZcam (40 ms integration time), is tightly linked to the minimal microbolometer temperature elevation and stabilization time response τ, evaluated to 10 ms for the CEA LETI-based TZcam [26]. Considering a pixel exposure time much lower than τ would then result in an approximated linear averaging over the integration time of a pixel and so a single frame. Based on the setup implementation geometry, for a typical galvanometer amplitude, with one mirror’s oscillating frequency of 50 Hz, the exposure time of a single central pixel is lower than 1ms considering the estimated beam diameter (see Figure 2b). The maximum mirror’s oscillating frequency of 130 Hz is then more than suitable for our averaging purposes in such a configuration, but is necessary for complex pattern generation.

From an implementation perspective, it is also interesting to note that the steered beam size in the image plane can be easily adjusted by tuning the distance between the focusing 45° OAPM and the imaging system, due to the slightly converging beam profile after the OAPM. This characteristic is another versatile aspect of the complete system that can be used to emphasize, either the homogeneity lighting, or the maximal reachable SNR.

## 3. Illumination Pattern

### 3.1. Principle and Simulations

Based on optimized 2D Lissajous curves surface scanning, several imaging systems use such trajectories for applications in endoscopy [27], microscopy [28] or for lidars [29], allowing to adjust a trade-off between pixel density, SNR and frame rate [30]. In the case of our real-time full-field imaging system, we implement an illumination pattern in the object plane controlled by two sinusoidal voltage signals fed into the galvanometer. Considering the linear voltage-to-angle conversion from each individual mirror of the galvanometer, the vertical and horizontal deviation angles from the OAPM aperture center, α(t) and β(t) respectively, are define as follow:

(1) $$\alpha \left(t\right)=A\;cos\left(2\pi F_{x}t+\phi \right)+\alpha_{0},$$

(2) $$\beta \left(t\right)=B\;cos\left(2\pi F_{y}t\right)+\beta_{0},$$

with *A* and *B*, the angular amplitudes, $F_{x}$ and $F_{y}$, the oscillation frequencies, ϕ, the relative temporal delay or phase introduced for pattern shaping improvement, and $\alpha_{0}$ and $\beta_{0}$, the offset angle respectively from the OAPM optical axis. A proper optical alignment ensures that, the latest are set to 0${}^{{}^{\circ}}$ when the angular pattern is centered on the aperture axis of the OAPM, and so when the THz illumination pattern is generated along the imaging optical axis.

For a generic OAPM geometry, performing in our case the angular-displacement conversion, the beam off-axis position r as a function of the paraboloid off-axis angle θ is given by:

(3) $$r\left(\theta \right)=2\;PFL\left(\frac{1-cos(\theta )}{sin(\theta )}\right),$$

with PFL, the OAPM parent focal length, equal to 152.4 mm in this setup. Induced by the OAPM 45${}^{{}^{\circ}}$ off-axis angle, therefore considering a relative horizontal displacement dictated by $r_{x}\;\left(\theta \right)=r\left(\theta \right)-r\left(45^{{}^{\circ}}\right)$, the horizontal displacement is brought to Equation (4) while the vertical distance to the OAPM center is directly given by the Equation (3) and leads to Equation (5). For simplification purposes, those equations have been evaluated assuming a punctual angular beam steering at the OAPM focal point, thus neglecting the spacing between the two galvanometer’s mirrors.

(4) $$X\left(t\right)=r\left(45^{{}^{\circ}}+A\;cos\left(2\pi F_{x}t+\phi \right)+\alpha_{0}\right)-r\left(45^{{}^{\circ}}\right),$$

(5) $$Y\left(t\right)=r\left(B\;cos\left(2\pi F_{y}t\right)+\beta_{0}\right).$$

Accounting for the galvanometer angular limitations $A_{max}$ and $B_{max}$, the achievable displacements are constrained to the interval [−58.68 mm; 67.93 mm] in the horizontal scanning direction and [−53.74 mm to 53.74 mm] for the vertical scanning, ensuring a total coverage of the OAPM aperture.

**Figure 3:**
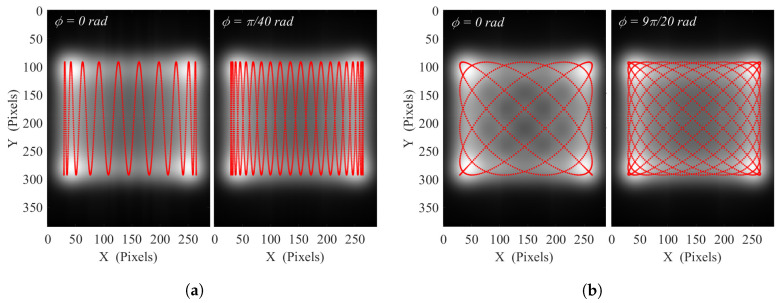
Simulated illumination patterns, resulting from the average of 2D Gaussian images with A = B = 6° and their relative closed Lissajous path ways (red) for (**a**) *γ* = 20 with *F_x_* = 6.25 Hz, *F_y_* = 125 Hz, during 160 ms and (**b**) *γ* = 20/18 with *F_x_* = 112.5 Hz, *F_y_* 125 Hz during 80 ms, at *ϕ* = 0 and ϕ=π2γ for both cases.

Equations (4) and (5) representing the time parametric equations related to the beam position at the object plan, the resulting beam trajectory is mapped out as a modified Lissajous pattern. The OAPM projection is represented by the r(θ) function and induces some distortion on the illumination beam. As a result of this specific Lissajous geometry, the horizontal and vertical frequency ratio γ=FyFx is required to be rational to form a closed scanning pattern [31]. This characteristic is necessary in our case to reach a repeatable illumination background. The chosen scanning oscillation frequencies will obviously impact the scanning pattern as well as its repetition rate that should fit the frame rate of the camera. In our implementation using the INO IRXCAM, a frame averaging factor of 8 is chosen on the camera, leading to a 6.25 FPS in order to suit the repetition rate of our chosen patterns (see Figure 3). Finally, the relative phase ϕ is a significant parameter to ensure the optimum homogeneity over the illuminated area on the sensor array. The least wanted configurations lead to the creation of degenerate paths in the case where ϕ = 0 mod(πγ) with the generation of a two-way path that will manifestly maximize the lighting heterogeneity. Contrariwise, the optimum homogeneity is reached for the most favorable spatial distribution of the path way, achieved for ϕ = π2γmod(πγ) [32].

Figure 3 displays, for *A* = *B* = $6^{{}^{\circ}}$, and the two relative phase values ϕ = 0 (degenerated pattern) and ϕ = π2γ (optimum spatial coverage), the simulations of two suitable distinct investigated patterns with (a) γ=20 at $F_{x}=6.25$ Hz, $F_{y}=125$ Hz, mimicking a smoothed sinusoidal raster-scanning illumination method, and (b) γ=20/18 at $F_{x}=112.5$ Hz, $F_{y}=125$ Hz, depicting a less intuitive pattern. To simulate the terahertz illumination on the sensors array, the 2D Gaussian function from the still mode image (Figure 2b) is averaged on every positions of the closed Lissajous path way in the image plane, considering the x0.22 lens magnification factor. The simulation spatial resolution is set to be the 35 μm array pitch. The temporal resolution is set to 50 μs for proper scanning sampling while a duration of 160 ms for γ = 20, and 80 ms for γ = 2018, ensures to complete the closed Lissajous curve scan for any relative phase ϕ. The animated pattern constructions, for those γ, ϕ and various FWHM can be visualized in the Supplementary Materials (Video S2).

As expected, the chosen relative phase and frequency pairs, and so γ Lissajous factor, are impacting the scanning pattern as well as the resulting homogeneity. To evaluate such illumination heterogeneity, which represents an important criterion for imaging quality, a calculation of the coefficient of variation ($c_{V}$) on the illuminated area is used and defined as the ratio of the standard deviation σ by the mean value μ, cV=σμ. In order to focus on the illuminated area only, μ and $c_{V}$ are calculated, for each amplitude angle, from image sections delimited by the maximal and minimal achievable center beam positions.

**Figure 4:**
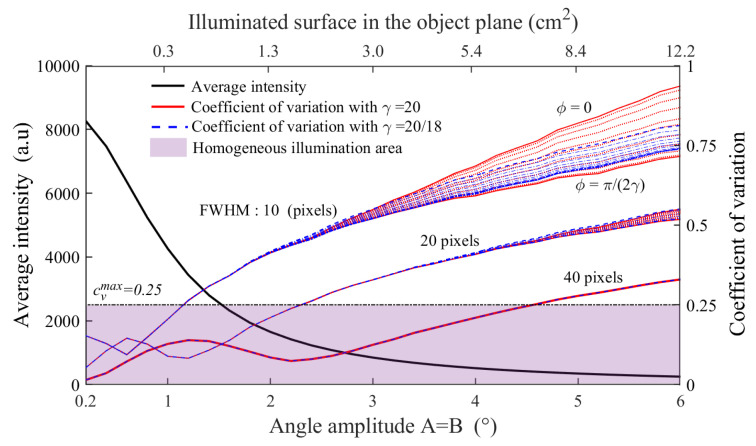
Simulated pattern average intensity, coefficient of variation and illuminated area in the object plane as a function of the oscillation amplitude angle, the beam diameter (FWHM) and the relative phase ϕ at γ=20 and γ=20/18.

In the ideal case of a perfectly homogeneous illumination, such as for single point raster-scanning, the coefficient of variation is equal to 0. This reference imaging method displays very weak signal variations, mainly induced by the source and detector noise, with typical 70 dB measurement dynamics. Considering a square command (A=B), Figure 4 displays, for a given γ Lissajous coefficient value, the simulated evolution of the average intensity (left axis) and the heterogeneity criterion, $c_{V}$ (right axis) as a function of the angular amplitude from 0.2${}^{{}^{\circ}}$ to 6${}^{{}^{\circ}}$. The corresponding illuminated surface in the object plane is also given for six different amplitude angles, ranging from 0.3 to 12.2 cm${}^{2}$. In addition, $c_{V}$ is calculated for varying relative phases ϕ from 0 to π2γ, as well as for three beam FWHM of 10, 20 and 40 pixels, corresponding to the following beams diameters in the object plane: 1.6, 3.2 and 6.4 mm respectively.

On one hand, the higher the amplitude, the larger the illuminated area. As a consequence, due to the limited emission power, and so accounting for the quadratic drop of the power density, the average intensity naturally decreases with the amplitude. On the other hand, the heterogeneity criterion $c_{V}$, calculated for various FWHM, exhibits a non-monotonous evolution, with an optimum located at oscillation amplitudes corresponding to pattern lobe size equal to the still beam FWHM, 2.3${}^{{}^{\circ}}$ in the case of the used 40 pixels FWHM beam. $c_{V}$ is then followed by an expected monotonous increase. In addition, the predominance concerning the impact of the relative phase as well as the choice of the γ Lissajous factor is noticeable for small scanning beam diameters and, considering a system scaling, would become for larger scanning amplitude with larger beams. Nevertheless, for our implemented 40 pixels FWHM beam diameter, the impact of the relative phase and the Lissajous pattern factor γ on $c_{V}$ remains negligible. Considering an acceptable homogeneous illumination for $c_{V}<0.25$ [33], an amplitude oscillation angle up to *A* = *B* = 4.4 ${}^{{}^{\circ}}$ is achievable.

### 3.2. Experimental Illumination

**Figure 5:**
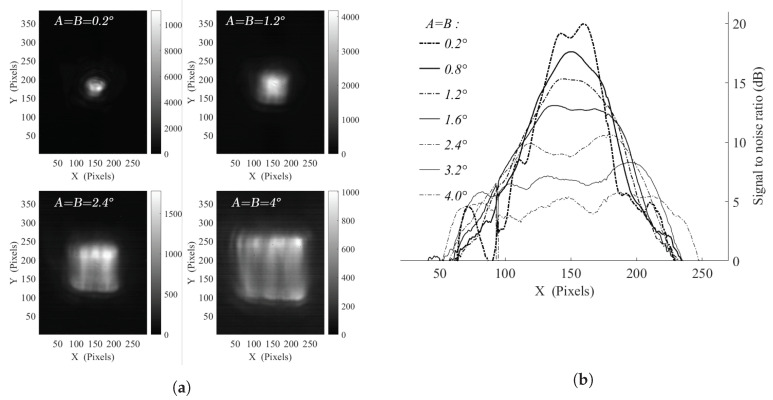
(**a**) Experimental illumination characterisations for *γ* = 20 with *F_x_* = 6.25 Hz, *F_y_* = 125 Hz and varying A = B = 0.2°; 1.2°; 2.4°; 4.0° illumination patterns; (**b**) SNR level evolution profiles along horizontal direction as a function of the driving amplitude for the generation of square patterns from A = B = 0.2° to 4.0°.

Experimental characterisations (see Figure 5) have been performed and corroborate the consistency of the simulations, particularly, the impact of modifying the size of the illuminated area. Figure 5a puts forward the experimental recorded illuminations for square driving signals at different scanning pattern amplitudes. Due to the low beam displacement, circular interference fringes are still noticeable at low oscillation amplitudes, up to *A* = *B* = 0.2${}^{{}^{\circ}}$. Besides, at high amplitudes, a slight distortion is induced by the OAPM projection. Figure 5b displays the horizontal cut of the illumination area which depicts a gradual decrease of the signal-to-noise ratio, in adequacy with the simulation results demonstrated in Figure 4b, while the homogeneity is impacted as well. The optimum homogeneity level, along with an adequate illuminated area, is reached as expected for *A* and *B* in between 1.6${}^{{}^{\circ}}$ and 2.4${}^{{}^{\circ}}$ for the 40 pixels FWHM beam setup. Moreover, in comparison with the conducted simulations, the non-perfectly Gaussian profile of the still beam (see Figure 2b) induces some ineluctable artifacts, impacting the achieved homogeneity.

## 4. Imaging Capabilities

With an appropriate fast beam steering in regard to the camera response time, a more homogeneous lighting is achieved and allows for the generation of versatile illumination patterns, suitable for imaging purposes. Thanks to the penetrability of THz waves in various dielectric materials, transmission imaging can offer a very interesting tool for non-destructive testing applications. The previously described setup (see Figure 1) is implemented for transmission imaging, but can easily be converted in reflection configuration by placing the camera next to the $45^{{}^{\circ}}$ OAPM, avoiding the insertion of a beam-splitter.

### 4.1. Real-Time Adjustable Imaging

The previously depicted Lissajous patterns ensure, considering an adequate selection of the oscillation amplitudes *A* and *B* at a given γ and ϕ, an optimised illumination of the interest area of a sample, tailored to its dimensions.

**Figure 6:**
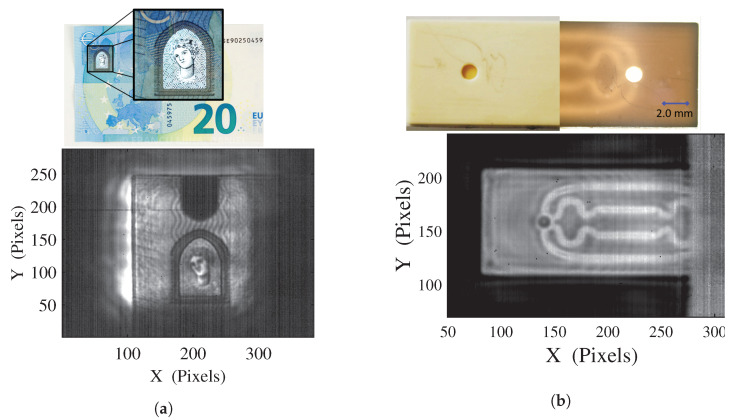
(**a**) Transmission image of a 20€ bill holographic strip at 2.5 THz, (**b**) Photograph, optical transmission image and 2.5 THz absorbance image of a 3D printed alumina-based ceramic micro-fluidic chip.

From this adequate illumination setup, optimized imaging capabilities can be expected. Figure 6a displays the obtained raw image of a part of holographic strip from a 20 € bill with galvanometer amplitude *A* = *B* = 4${}^{{}^{\circ}}$ for a 30 × 30 mm${}^{2}$ illuminated area, recorded at 6.25 Hz and using the same scanning pattern as for Figure 3a. Assessed through measurements on 1951 USAF test charts, a resolution of 250 μm is achieved at 2.5 THz (λ=120μm), limited by the numerical aperture of the optimized aspherical lens design (40 mm-f/0.83-x0.22).

In transmission configuration, further image processing steps allow improved visualization capabilities. Especially, in order to get rid of the illumination heterogeneity, a background image subtraction can be performed and gives access to a normalized transmission image of the sample. Furthermore, considering the exponential Beer-Lambert law, an application of a logarithmic scale to the transmission image leads to absorbance images in decibels (see Equation (6)) and enables high dynamic range visualization.

(6) $$Abs=-10log_{10}\left(\frac{\Phi_{obj}}{\Phi_{back}}\right)$$

with $\Phi_{back}$, the received optical flux from the background illumination without sample, and $\Phi_{obj}$, the received optical flux in presence of the investigated object in the illumination path. It should be noted that the flux difference in presence of the sample can be induced by the material absorption, interfaces reflections, diffusion and beam refraction.

Figure 6b displays the processed absorbance image of a 3D printed alumina-based ceramic chip, revealing internal 500 μm micro-fluidic channels, with optimized galvanometer amplitude of *A* = 2${}^{{}^{\circ}}$ and *B* = 4${}^{{}^{\circ}}$, recorded at 6.25 Hz, leading to a 160 ms exposure time, and using the same scanning pattern as Figure 3b. To perform such a processing, a background image of the illumination beam should be acquired, thus, requiring for a stable illumination. It is important to note that, as shown in Figure 2a, this image processing would be impracticable without an efficient averaging of the interference fringes induced by the optical path differences in the sample.

### 4.2. Enhanced Approaches for Versatile Imaging

Besides the improved capabilities of the galvanometer implementation compared to other homogenisation technique such as the optical scrambler, and thanks to the adaptable aspect of this technique, further improvements can be provided through different illuminations techniques in order to overcome the limited emission power of the source.

#### 4.2.1. Multi-Exposure Iimaging

**Figure 7:**
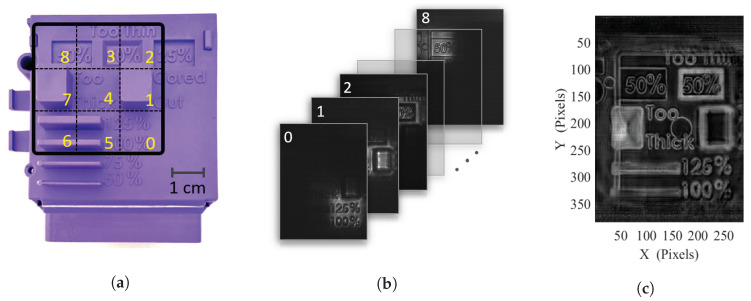
(**a**) Picture of the high density polyethylene sample with visualization of the sub-illuminated areas, (**b**) stack of the unprocessed recorded transmission sub-images at 2.5 THz, (**c**) absorbance reconstructed multi-exposure image.

Indeed, to overcome the quadratic SNR decrease in regard to the oscillation amplitude, a first solution, comparable to expanded raster scanning, consists in illuminating successively smaller areas of the sample. Along with the acquisition of such sub-images with adjacent limited illuminations areas, an improved power density leading to a better localized SNR is then achieved. This sequencing can indeed be performed by considering a smart combination of the oscillation amplitudes *A* and *B*, and a sequence of adequate offsets $\alpha_{0}$ and $\beta_{0}$ to illuminate a different sample portion for each frame (see Figure 7b). Combined with the background illumination measurement for the lighting pattern of each sub figure, an adequate processing is then practicable. Supplementary Video S3 displays the real-time sequence of absorbance sub figures.

A simple sum or averaging of the complete stack of images would grant a proper reconstruction of the image, but would result in the integration of the noise included in each non-illuminated area. A more appropriate approach consists in carefully selecting the sub-image portion where its illumination is maximal. Such a selection process, by considering the illumination background level of each sub-image, ensures an automated area selection on each sub-image for the final reconstruction, thus only considering the high SNR sections of each image. Further processing for absorbance image generation can then be performed to reach an improved SNR image, based on multiple smaller illumination area images (see Figure 7c).

For this implementation on the 2.5 THz setup, a high-density polyethylene (HDPE) sample is investigated (see Figure 7a), with a 3 × 3 illumination subdivision. Square subdivisions have been considered with *A* = *B* = 2${}^{{}^{\circ}}$ oscillation amplitudes and $\alpha_{0}$ and $\beta_{0}$ offsets combinations in { –4${}^{{}^{\circ}}$; 0${}^{{}^{\circ}}$; 4${}^{{}^{\circ}}$ } to form the 9 adjacent sub-illumination areas. For such a 3 × 3 subdivision configuration, a 10 dB SNR improvement is achieved and the effective illumination area is divided by a factor of 9 compared with a single exposure full field illumination, thus requiring a total image exposure of under 2 s. Larger subdivision levels proved to further improve SNR comparisons, being helpful for highly absorbent objects, larger illumination areas and for lower power sources or less sensitive detectors. Nevertheless, doing so will increase the required number of frame for a full acquisition, thus reducing the effective imaging frame rate. A frame rate/SNR trade-off can then be adequately selected, depending on the sample geometry and the desired recording rate.

With efforts toward industrial non-destructive testing, such sequential illumination technique could allow for selective screening of sample’s interest area, to ensure optimum detection performances with high cadences while offering unrivaled flexibility.

#### 4.2.2. Linear Illumination Object Scanning

Another similar implementation, especially suitable for inline inspection, or for sample with high form factors, consists in the generation of a linear illumination beam, used for scanning through an orthogonal displacement of the object.

Such a continuous displacement of the object ensures the capability of simply concatenating part of the successive images to obtain a reconstruction of the object. The width of this portion of image to be considered in pixel count $N_{pix}$ is obviously linked to the object displacement speed, $v_{o}$, the camera frame rate, FPS, as well as the lens magnification, *M*, and the detector’s pitch, $\delta_{pix}$ as follows:

(7) $$N_{pix}=\frac{v_{o}M}{\delta_{pix}FPS}$$

A spatial sub-sampling of the image is nevertheless necessary to improve the reconstruction precision, depending on the selected object displacement speed. Furthermore, considering the width of the linear illumination pattern in comparison with the object displacement speed, a shifted averaging over a few frames is performed in order to further improve the obtained SNR, and dampen any time related fluctuation.

However, due to the limitation of the imaging frame rate caped by the oscillating frequency of the galvanometer in order to keep a repeatable and homogeneous illumination, the object displacement speed remains also limited in order to avoid any motion-induced blur.

**Figure 8:**
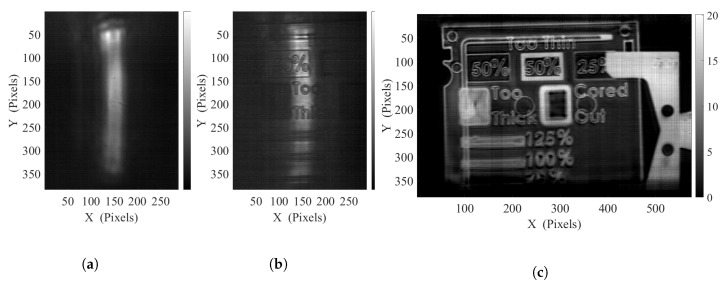
(**a**) Background linear illumination pattern, (**b**) single frame image corresponding to a narrow part of the sample, (**c**) reconstructed absorbance image of the scanned object using a sub-sampling factor of 4, and shifted averaging over 5 frames for an object displacement of 20 mm s^−1^.

In this implementation, the same HDPE sample presented in Figure 7a has been investigated on the same setup. A full width illumination of the sensor have been achieved for this unidirectional lighting, with *B* = $8^{{}^{\circ}}$ and *A* = $0^{{}^{\circ}}$ and with an oscillating frequency of $F_{y}=125$ Hz. The Figure 8a,b, represents respectively the unidirectional background illumination and a given frame of the object scan. The unidirectional oscillation set to the galvanometer ensures a faster pattern repetition rate, providing a faster accessible frame rate, set to 25 FPS (averaging factor of 2) for this measurement, allowing an object displacement at the speed of 20 mm s${}^{-1}$ and so a measurement time under 4 seconds for the full sample scan. Reminiscent coherence artifacts remain noticeable in the vicinity of the edges as well as on the side of the illumination patterns due to the more regular nature of this displacement scheme. Nevertheless, for the object reconstruction, only the homogenized part of the pattern is considered. The video-rate displayed linear scanning can be visualized in the Supplementary Content S4, while the resulting object reconstruction is given in Figure 8c.

### 4.3. Illustration to 3D Tomographic Imaging Operating with a Limited Power Source

**Figure 9:**
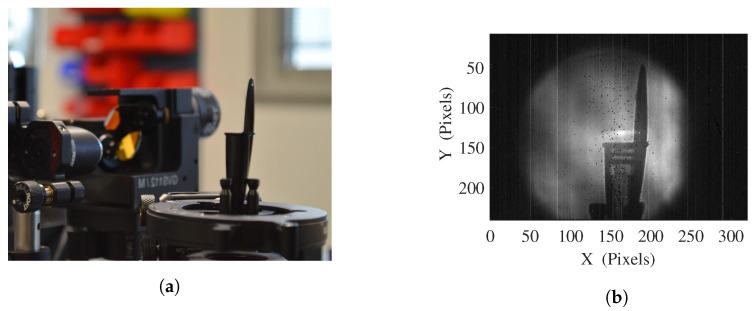
(**a**) Picture of the black polypropylene pen cap mounted on its holder (**b**) A 2D projection of the sample at 3.78 THz resulting from the multi-exposure processing using a combination of 49 illumination patterns.

Finally, the versatility of the presented imaging system enables faster 3D terahertz computed tomographic reconstructions. Tomography, first applied on X-ray images for medical and NDT industrial purposes, processes a series of 2D transmission projections of the object under various viewing angles, representing sinograms. Based on the inverse Radon transform, tomography algorithms consider any contrast in the sinogram as absorption through the sample. The outcome is a 3D depiction of the entire sample. Since its first application to terahertz images [34], numerous studies demonstrated improvements and adaptations of the algorithm to the specificity of terahertz ray [35,36]. However, as far as we know, none of those reconstructions have been performed using such a fast and adaptable terahertz imaging system. We propose to apply the terahertz-optimized Ordered Subset Convex algorithm (OSC) [37] on a sinogram provided by the newly presented imaging system, exploiting the TZcam illuminated at 3.78 THz by the TC100. Because of the limited radiation power, a drastic reduction of the illuminated sample area is required. In order to provide complete projections of the entire sample, the multi-exposure imaging technique, presented in the Section 4.2.1, is exploited using a 7 × 7 illumination subdivision of the field with a 2 FPS video rate instead of the available 25 FPS, leading to a 25 s full frame acquisition time. The sample, mounted on one rotating motor, is a black pen cap (see Figure 9a) chosen for its transparency at 3.78 THz. Thanks to the synchronisation between the source, the galvanometer, the rotating motor and the camera, the system is autonomously providing the projection from $0^{{}^{\circ}}$ to $360^{{}^{\circ}}$.

The 2D projection, displayed in Figure 9b, results from the multi-exposure imaging processing with an offset combination of $\alpha_{0}$ and $\beta_{0}$ in {$-6^{{}^{\circ}}$; $-4^{{}^{\circ}}$; $-2^{{}^{\circ}}$; $0^{{}^{\circ}}$; $2^{{}^{\circ}}$; $4^{{}^{\circ}}$; $6^{{}^{\circ}}$ }${}^{2}$ with *A* = *B* = $1^{{}^{\circ}}$, $F_{x}$ = 25 Hz and $F_{y}$ = 125 Hz. Unlike the precedent imaging system, the illuminated area on the sensors array is limited by a reduced entrance pupil diameter in the illumination system. Besides, no interference fringe is observed despite the small amplitude angle.

**Figure 10:**
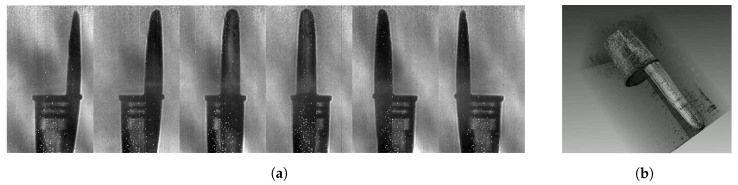
(**a**) Selection of 6 cropped and centered 2D projections of the sample among the 36 acquired from 0° to 175° (**b**) 3D isometric visualization of the pen cap reconstructed by terahertz computed tomography.

The multi-exposure procedure is repeated 36 times to provide a sinogram of the sample from $0^{{}^{\circ}}$ to $175^{{}^{\circ}}$ with a $5^{{}^{\circ}}$ step angle in under 15 min. In order to ensure a proper tomographic reconstruction, additional image processing is required. The projections are divided by the illumination background, leading to a stack of normalized transmission images. Indeed, the algorithm used for tomography includes the exponential absorption of Beer-Lambert law. An image centering is required as well to ensure a proper positioning of the rotation axis. Figure 10a displays a selection of 6 transmission images, representing a 34 × 20 mm${}^{2}$ area in the object plane for various viewing angles. Despite the image normalization, the resulting background is not perfectly uniform and varies for each projection angle due to a desynchronization between the camera read-out and the illumination Lissajous galvanometric beam steering. Nevertheless, the inside structure of the pen cap is easily observable through the polypropylene material, notably the two extruded bumps used to hold the pen closed.

Figure 10b is a 3D isometric visualization of the reconstructed pen cap, exploiting the OSC algorithm included in the software Noctylab [38]. Such a rendering is possible by the application of a transparency threshold on the linear gray-scale. Indeed, the 3D reconstructed object contains low absorption voxels that have to remain transparent for a proper rendering of the sample itself. A more complete 3D view is available in the Supplementary Video S5. Due to different factors, such as the non homogeneous illumination as well as the refraction and reflection losses at the air/PP interfaces that are not considered in the algorithm, the final reconstruction displays various external artifacts and some inconsistencies on the internal structure. Nevertheless, the sample is properly reconstructed with the sharp sub-milimetric optical resolution expected at 3.78 THz, with only 100μW available power for a 5 × 5 cm illumination field.

## 5. Discussion

The presented results emphasize the imaging capabilities and the versatility of this galvanometric illumination system in the terahertz domain. Enabling full-field video rate through near raster-scanning illumination, this hybrid imaging system has the capability to adapt its performances to the dimensions and the nature of the object under investigation. The detailed experimental results illustrate this versatility through the convenient modification of the angle amplitude oscillation, to ensure an adaptability of the average dynamic range, suiting the sample’s dimension. In addition, combining this lighting strategy with a beam raster-scanning, larger size images were performed, conserving an adequate SNR. This proof of concept can suit the required imaging rate for NDT applications in 2D as well as in 3D.

Compared with the conventional raster-scanning imaging process, the mandatory acquisition time is drastically reduced. Typically, considering the same radiation power source, several hours are required for a properer image acquisition, and up to days to complete tomographic reconstructions, while optimized video rate recording is achieved in this configuration. This significant speed-up is ensured by the efficiency of THz lighting steering, where no movement of the imager or the sample is required, as well as the high sensitivity and low Noise Equivalent Power of the microbolometer arrays in comparison with single pixels detectors [39].

Concerning the illumination homogeneity, we demonstrated the ability to strongly reduce the impact of the interference fringes on the final image. Nevertheless, reminiscences of these fringes remain noticeable at the edges of the pattern, where the illumination spot trajectory on the sensor array undergoes a slowdown to reach a quasi static position before speeding up anew. This artifact raises the predominant aspect of the beam steering phase, since it is especially noticeable for degenerate Lissajous patterns for which an illumination beam halt occurs. Besides, the experimental observed homogeneity is affected by the OAPM projection as well as the non-symmetrical static illumination spot. Finally, the limited mechanical frequency oscillation of the galvanometer mirrors, in collaboration with the camera timeout inbetween each recorded frame, are impacting the illumination heterogeneity, resulting in repeatable under-illuminated clusters. At equivalent γ value, a faster galvanometer, able to loop over the Lissajous curve during the typical bolometer response time, would lead to an improvement in homogeneity.

More generally, the resulting illumination homogeneity remains far from the capabilities of raster-scanning techniques. Nevertheless, owing to the recording of the repeatable illumination background, no visible impact of the interference fringes is ensured through simplified data processing steps in order to reach high quality transmission or absorption images of the investigated samples while ensuring video rate recording.

## 6. Conclusions

In this study, we presented an innovative imaging solution, with a strong focus on the illumination techniques, able to provide high-quality far-field video-rate visualizations in the terahertz domain, in order to see through interesting optically opaque material. As a result of the versatility of this solution, we overcome both the coherency issue inherent to the sources as well as their limited radiation power. Moreover, this adaptability allows terahertz imaging in different configurations for transmission and reflection, and for various object geometries via multiple imaging schemes like inline scanning (1D), multi-exposure imaging (2D) or tomography reconstruction (3D). Along with the effort to persistently increase the maximal radiation output power of emitters such as infrared QCL pumped molecular laser [40] or gyrotrons [41], we bring forward terahertz imaging as a powerful tool for non-destructive testing applications. Current work is targeting the transposition of this system to lower frequency domain to benefit from the higher material penetrability while keeping a suitable resolution.

## Supplementary Materials

The following are available at https://www.mdpi.com/1424-8220/20/14/3993/s1. Video S1: Illustration of the interference fringes shift in a basic transmission mode implemented focal plane array imaging setup while rotating a phase object illuminated by a coherent source at 2.5 THz. Video S2: Time-resolved constructions of Lissajous curves and corresponding simulated illumination patterns for various γ, ϕ and FWHM. Video S3: Real-time sequence of absorbance sub figures for the multi-exposure imaging method. Video S4: Real-time sequence of the displayed absorbance linear scanning. Video S5: 3D animated visualization of the reconstructed pen cap.

## Abbreviations

The following abbreviations are used in this manuscript:

SNR	Signal to Noise Ratio
FPS	Frames Per Second
OAPM	Off-Axis Parabolic Mirror
FWHM	Full Width at Half Maximum
NEP	Noise Equivalent Power
NDT	Non Destructive Technique
OSC	Ordered Subset Convex
QCL	Quantum Cascade Laser
HDPE	High Density Polyethylene
PP	Polypropylene
