# Supplementary figures and images for: A Versatile Illumination System for Real-Time Terahertz Imaging
Source: Sensors (Basel). 2020 Jul 17;20(14):3993. doi: 10.3390/s20143993 (PMC7412008; doi:10.3390/s20143993)

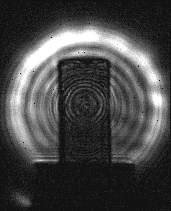

Supplement: Supplementary file 1 [file sensors-20-03993-s001.zip › Supplementary materials/Video S1.gif]

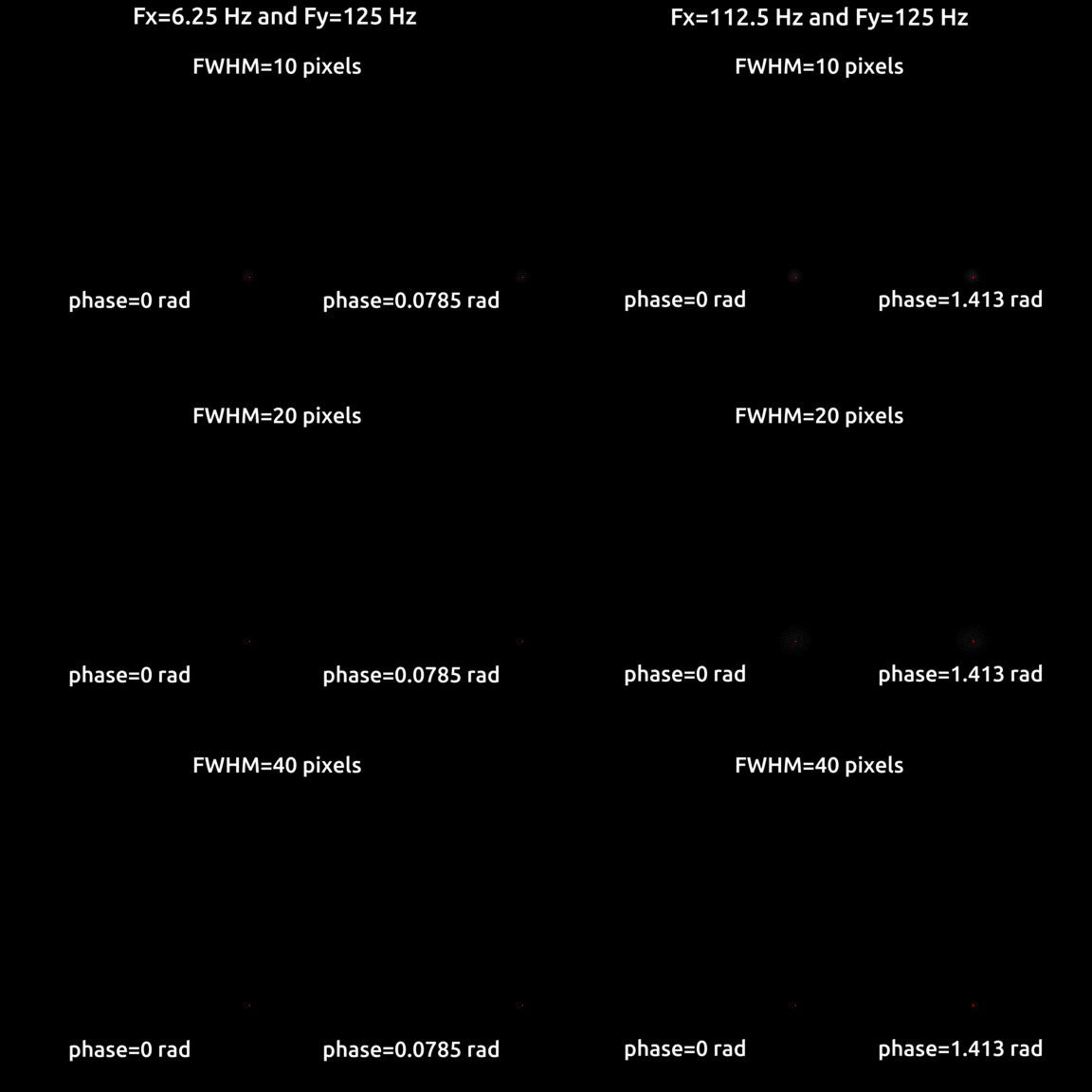

Supplement: Supplementary file 1 [file sensors-20-03993-s001.zip › Supplementary materials/Video S2.gif]

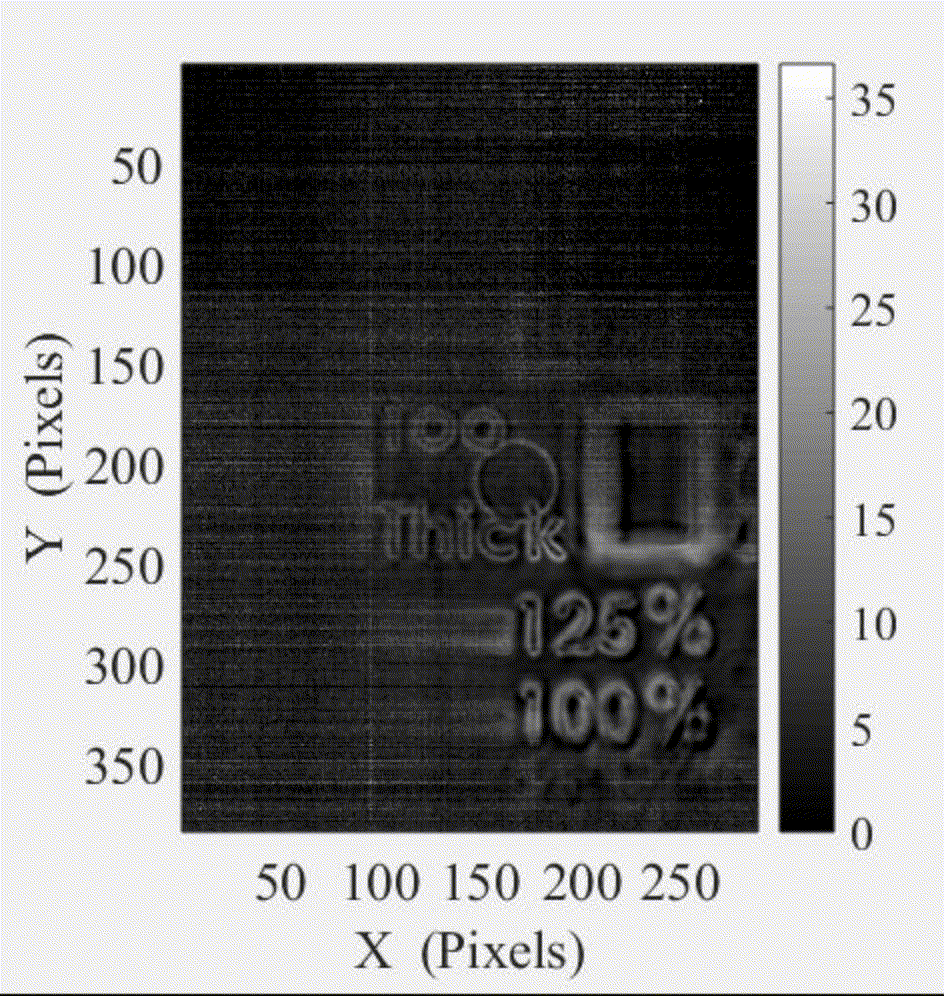

Supplement: Supplementary file 1 [file sensors-20-03993-s001.zip › Supplementary materials/Video S3.gif]

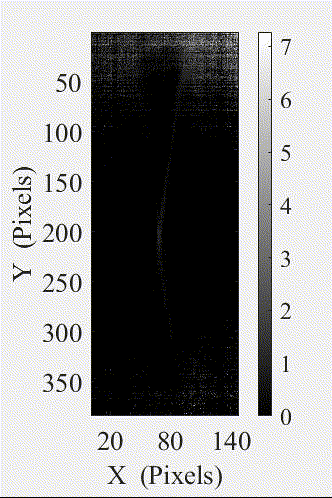

Supplement: Supplementary file 1 [file sensors-20-03993-s001.zip › Supplementary materials/Video S4.gif]

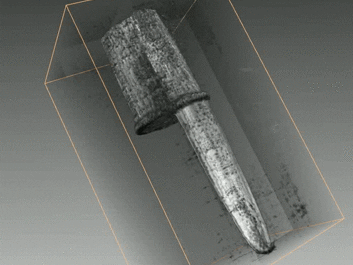

Supplement: Supplementary file 1 [file sensors-20-03993-s001.zip › Supplementary materials/Video S5.gif]
